# Patterns of orchid bee species diversity and turnover among forested plateaus of central Amazonia

**DOI:** 10.1371/journal.pone.0175884

**Published:** 2017-04-14

**Authors:** Yasmine Antonini, Carolina de Barros Machado, Pedro Manoel Galetti, Marcio Oliveira, Rodolfo Dirzo, Geraldo Wilson Fernandes

**Affiliations:** 1 Departamento de Biodiversidade Evolução e Meio Ambiente—Universidade Federal de Ouro Preto–Campus Morro do Cruzeiro, s/n, Ouro Preto, MG, Brazil; 2 Biology Department, Stanford University–Sierra Mall. Stanford, CA, United States of America; 3 Departamento de Genética e Evolução, Universidade Federal de São Carlos, Via Washington Luis Km 235, São Carlos, SP, Brazil; 4 Instituto Nacional de Pesquisas da Amazônia, Manaus, AM, Brazil; 5 Departamento de Biologia Geral, Universidade Federal de Minas Gerais–Av Antonio Carlos 6627 –Belo Horizonte, MG, Brazil; Universidade de Sao Paulo Faculdade de Filosofia Ciencias e Letras de Ribeirao Preto, BRAZIL

## Abstract

The knowledge of spatial pattern and geographic beta-diversity is of great importance for biodiversity conservation and interpreting ecological information. Tropical forests, especially the Amazon Rainforest, are well known for their high species richness and low similarity in species composition between sites, both at local and regional scales. We aimed to determine the effect and relative importance of area, isolation and climate on species richness and turnover in orchid bee assemblages among plateaus in central Brazilian Amazonia. Variance partitioning techniques were applied to assess the relative effects of spatial and environmental variables on bee species richness, phylogeny and composition. We hypothesized that greater abundance and richness of orchid bees would be found on larger plateaus, with a set of core species occurring on all of them. We also hypothesized that smaller plateaus would possess lower phylogenetic diversity. We found 55 bee species distributed along the nine sampling sites (plateaus) with 17 of them being singletons. There was a significant decrease in species richness with decreasing size of plateaus, and a significant decrease in the similarity in species composition with greater distance and climatic variation among sampling sites. Phylogenetic diversity varied among the sampling sites but was directly related to species richness. Although not significantly related to plateau area, smaller or larger PD_Faith_ were observed in the smallest and the largest plateaus, respectively.

## Introduction

The knowledge of spatial pattern of beta-diversity is of great importance for practical biodiversity conservation and interpreting ecological information [1]. Studies of patterns of diversity in the Amazon–the largest and richest rainforest biome in the world–have traditionally focused on a restricted number of well-known taxa, such as plants and vertebrates [2–4]. To our knowledge, few studies have been conducted focusing on systematic inventory involving insects [5–8]. This is surprising given that insects comprise the largest portion of Amazonian biodiversity and the vast majority of its animal biomass [9].

Tropical forests, especially the Amazon Rainforest, are well known for their high species richness and low degree of similarity in species composition among sites, both on local and regional scales [10, 1]; in other words, there are few common species among sites. Differences in species richness and composition among sites is attributed to beta diversity (or high species turnover among sites), as a result of heterogeneity in habitats and species dispersal limits [11,1, 12]. On a broad scale, contemporary spatial patterns of species richness are influenced by, among other factors, current climate [13–15], as well as historical events that influenced the geographical origins and distribution of clades [16–17].

Although central Amazonia is known to be primarily composed of lowland rain forests, the region of Saracá-Taquera, in the State of Pará, is of particular interest due to its unique topography in the form of plateaus that reach 200 meters in elevation [18].

Unfortunately, these plateaus have been undergoing rapid and intense destruction due to bauxite mining, land development, and logging. Therefore, understanding the diversity of organisms living among the plateaus is critical for developing proper strategies for species conservation and management, as well as for understanding the resilience of the entire ecosystem. Otherwise, because the plateaus, hidden in the middle of the central Amazon, must have unique species composition and structure, the outcome in terms of biodiversity and ecosystem services loss may have been very high and neglected up to now. Furthermore, the understanding of native bee fauna is of much interest as there is a general trend of pollinator decrease in many parts of the world.

Orchid bees are a promising group for understanding the patterns that drive beta diversity in the Amazon region. Orchid bees are known to be more diverse and abundant in forested areas closer to the Equator [19], where they are regarded as key pollinators [20–21].

Most orchid bee species are dependent on tropical rainforests, which guarantee a continuous supply of nectar and pollen as well as different odoriferous substances used in courtship displays [21]. Also, being agile and far-flying organisms, orchid bees are able to travel many kilometers daily in search of food and fragrances [22], and thus favoring the colonization of new environments. In studies of Amazonian biodiversity, the taxonomy of Amazonian orchid bees is well established [23,24], aiding their identification to the species level. On the other hand, knowledge about trends in the diversity of orchid bees is limited due to a lack of studies in the Amazonian region e.g. [23–26; 6–7; 27].

Here we evaluate the influence and relative importance of spatial and environmental factors in explaining variation in species richness and composition of orchid bee assemblages of nine plateaus in the forest of central Amazonian in Brazil. We tested the hypothesis that greater abundance and richness of orchid bee species will be found on larger plateaus. We also predicted that a set of specialists species would be present on all plateaus, and that smaller plateaus would present lower phylogenetic diversity of orchid bees following the lower species richness. Therefore, it is expected that the number of common species will be greater in large plateaus, while rare species would be absent from smaller plateaus.

## Methods

**Fig 1:**
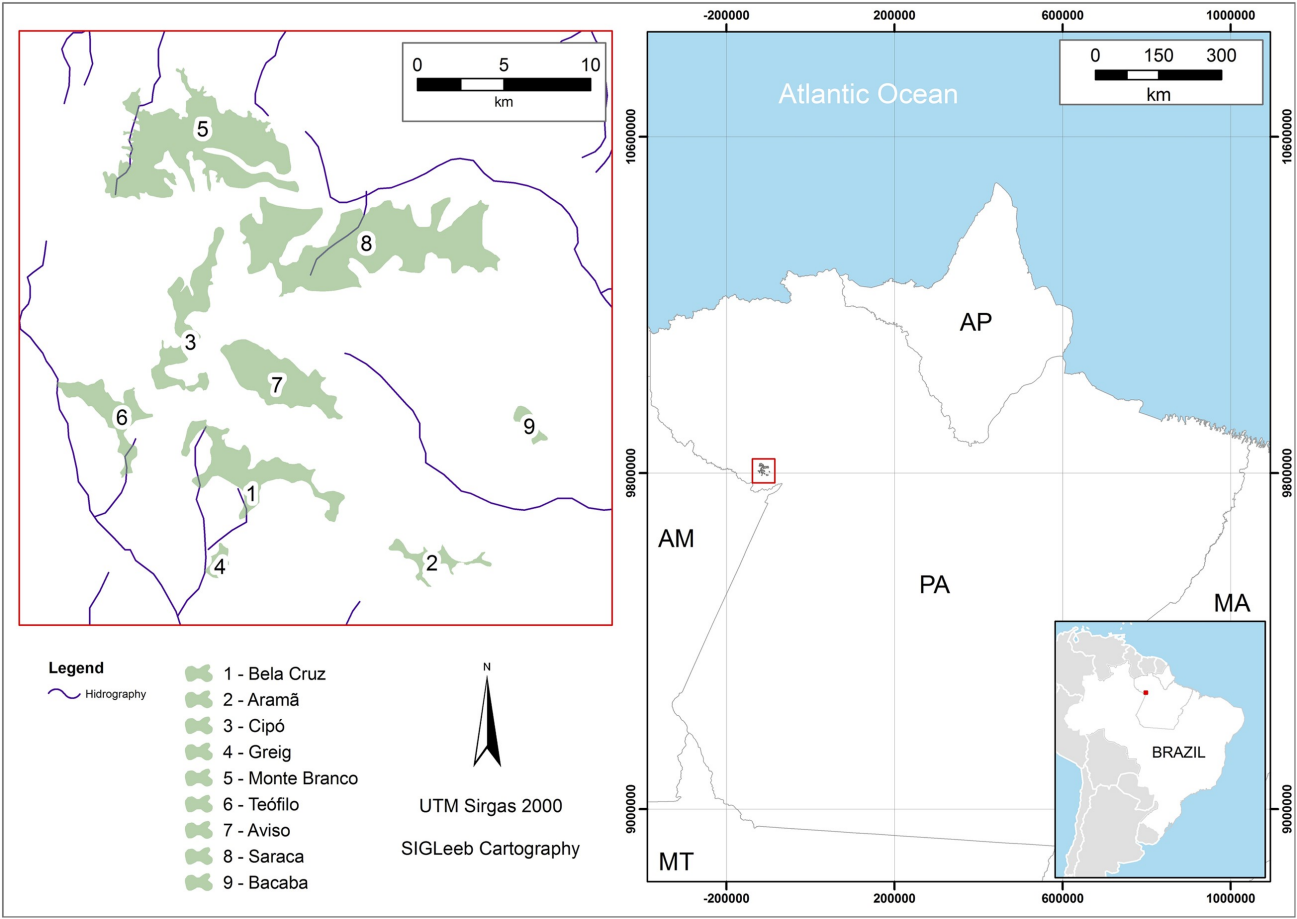
Map of the study area showing the location of our nine sampling sites in the FLONA Saracá-Taquera Brazilian Amazon.

The Floresta Nacional (FLONA) Sacará-Taquera in the municipality of Oriximiná, Pará, Brazil (Fig 1) possesses areas of relatively high elevation known as plateaus. These plateaus may reach 200 m a.s.l. [18] and are covered by dense and highly diverse Amazon Rainforest [28] (Fig 1). The climate of the region is Am Tropical with monsoons, according to the Köppen classification [29], with annual rainfall ranging from 2,200 mm to 2,500 mm and temperatures ranging from 20°C to 29°C.

Orchid bees were collected from nine plateaus scattered throughout the FLONA Saracá-Taquera (under IBAMA permission 085/2006), with distances between sampling sites ranging from 8 to 30 km (Fig 1). The sampled plateaus were Aramã (AR), Aviso (AV), Bacaba (BB), Bela Cruz (BC), Cipó (CP), Greig (GR), Monte Branco (MB), Saracá (SR) and Teófilo (TF). Bee sampling took place in 2006, 2007, and 2008 during both the dry and wet seasons. On each plateau, three “sampling plots” were established at least 2,000 m from each other in undisturbed forests (i.e. primary, non-successional forests), following Brasil [30].

To trap a high percentage of the orchid bee species occurring at a given site, bees were baited with specialized scent compounds comprised of one of the following substances known or believed to be attractive to orchid bees: 1,8-cineole, b-ionone, benzyl acetate, eugenol, methyl benzoate, trans-methyl cinnamate, methyl salicylate, and vanillin [31]. Baits, comprised of cotton pellets, were hung 1.5 m above the ground and 20 m from one another, and were soaked with their respective essence every two hours. All specimens collected were deposited in the entomological collection of the Museu Paraense Emilio Goeldi in Belém, and in the invertebrate collection of INPA in Manaus, both in Brazil.

Information about the spatial location of bees (distance between sites, in km), elevation, average annual temperature, average annual precipitation, and potential of evapotranspiration was collected for each of the nine study plateaus. These climatic variables were previously shown to be of importance in affecting the large-scale distribution of orchid bees [6]. We used data from WorldClim [32] to determine the average annual rainfall and data from LAPIG (home page here) to determine the average evapotranspiration rate at each site.

We used non-metric multidimensional scaling (NMDS), an indirect gradient analysis, to describe and interpret the major gradients in the orchid bee community data. We estimated the level of compositional similarity between pairs of sampling sites using the Simpson Índex because it provides a good measure of beta diversity by focusing on compositional differences between sites independent of species-richness gradients [33] and, consequently, of variation in sampling effort among sites. Ordinations and inferential statistical tests were performed using the Statistica program. Only species present in at least two forest sites were included in this analysis, as rare species may distort the analysis and impede a reliable description of the main patterns of variation [5].

The relationship between geographic distance and similarity in species composition, also known as the distance-decay in similarity relationship [10], was analyzed using simple linear regression. Distance-decay plots were built in order to analyze variation in compositional similarity among pairs of sampling sites in relation to the geographic and climatic distances between sites [10]. The geographic matrix was built using the Euclidean distance between sites based on their distance in kilometers using the ArcGIS program.

The Euclidean distance was also used to build the climatic matrix, which contained information considering the average annual temperature, average annual precipitation, and precipitation seasonality of each site (given by the potential of evapotranspiration).

Before calculating the Euclidean distance between sites each variable was standardized so that all variables included in the analyses had a mean of 0 and a standard deviation of 1.

The significance of these relationships was evaluated using the Mantel test [34]. Before calculating the phylogenetic diversity, we conducted Bayesian phylogenetic inference based on mitochondrial (cytochrome c oxidase I, COI) and nuclear (elongation factor 1-a (EF1-α), arginine kinase (Argk) and RNA polymerase 142 II (Poll-II)) genes extracted from GenBank (S1 Table) [7, 35]. The dataset contained 39 taxa (4354 base pairs), which represent 73% of our collected species (or morphospecies).

Each of the four genes had separate model of nucleotide evolution estimated using the program jModeltest 2.1.4 [36] based on Bayesian information criterion (S2 Table).

The phylogenetic relationship reconstruction among orchid bees was conducted in MrBayes v3.2 [37]. Three heated chains and a single cold chain were used in the MCMC analysis and run was initiated with random tree. MCMC ran for 10 million generations sampled at every 1000 steps. The initial 1% of sampled topologies were discarded as burn-in. Proper mixing was verified with Tracer v1.5 [38], and an effective sample size (ESS) of 200 or higher was required for all parameters.

To measure phylogenetic diversity, we used two metrics: Faith’s phylogenetic diversity (PD_Faith_) [39] and mean pairwise phylogenetic distance weighted by species abundance (MPD) [40] in each study site. Both metrics were estimated using Picante (Phylocom integration, community analyses, null-models, traits and evolution) [41] package for R (http://www.Rproject.org). The PD_Faith_ has been defined as the sum of the branch lengths of the phylogenetic tree that connects all species from the community. On the other hand, MPD is the average of phylogenetic distances between all pairs of species in each community. The latter is mathematically less dependent on species richness and would, in principle, reduce statistical problems related to multicolinearity.

We also used linear regression to test for the existence of a positive relationship between phylogenetic diversity (PD_Faith_ and MPD) vs. plateau size, and phylogenetic diversity (PD_Faith_ and MPD) vs. orchid bee species richness.

## Results

**Fig 2:**
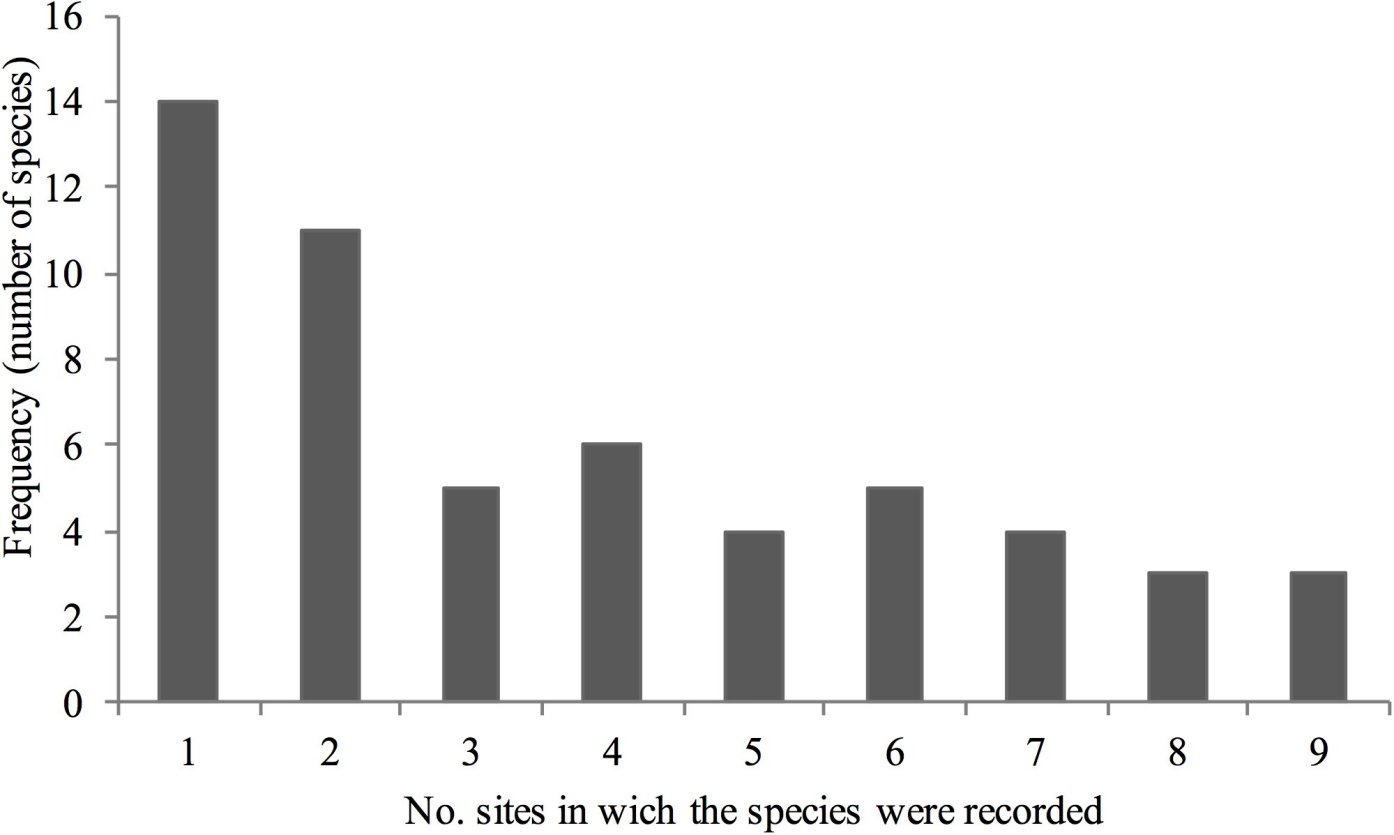
Number of Plateaus in which each of the 55 orchid-bee species found at FLONA Saracá-Taquera, Brazil, were recorded.

The total sampling effort resulted in 486 h of sampling and the capture of 1,673 male orchid bees belonging to four genera and at least 55 species (S2 Table). Twenty-six species (or morphospecies) were rare, being found on only one or two of our study plateaus (Fig 2).

**Fig 3:**
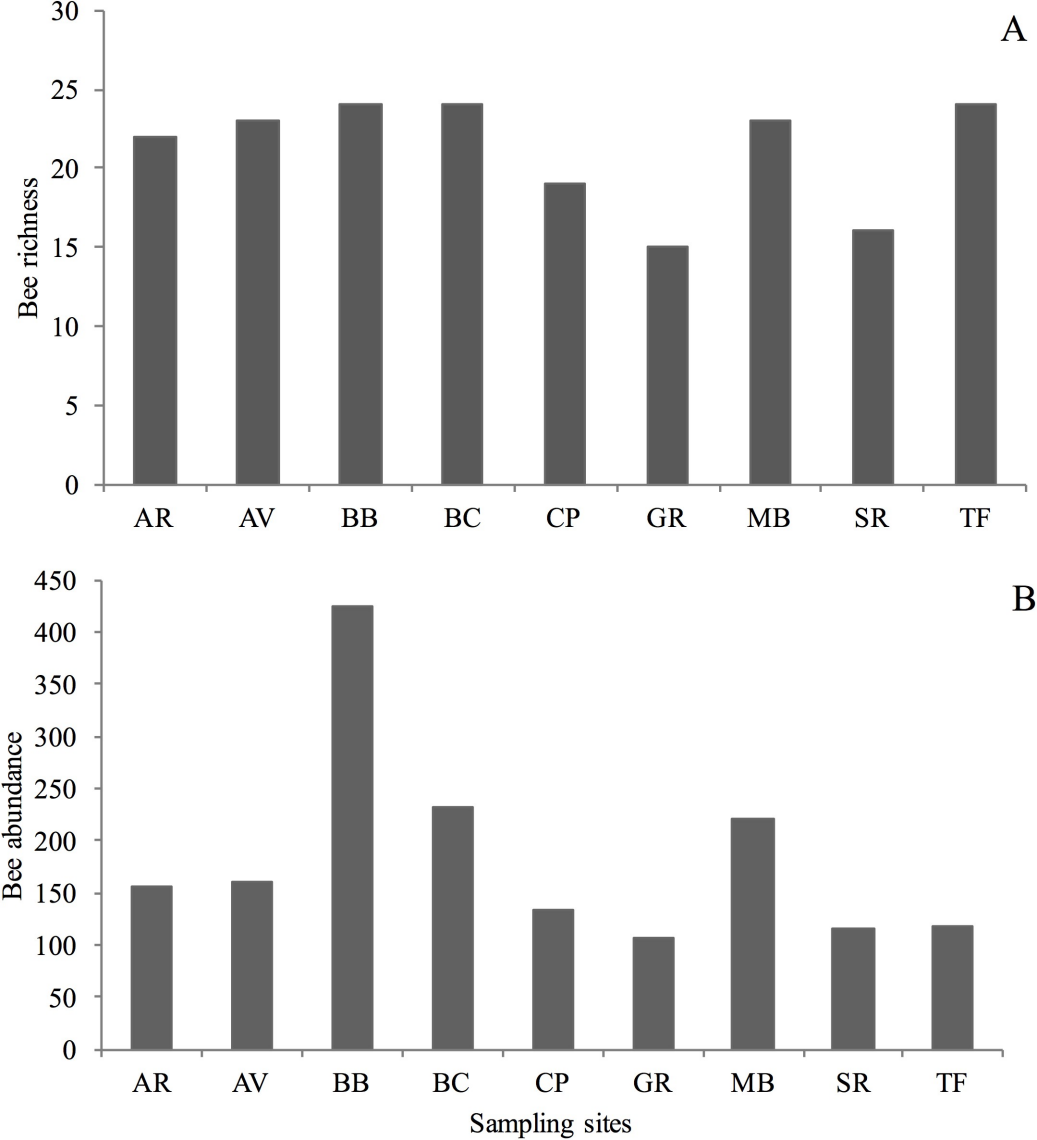
Orchid bee richness (A) and abundance (B) in each sampling site, FLONA Saracá-Taquera, Brazil. Codes are names of the plateaus, as in Fig 1.

Total orchid bee richness in the plateaus (including singletons) varied from 15 to 24 species, while bee abundance varied from 108 to 424, considering all species in all plateaus (Fig 3A and 3B and S2 Table). Only two species, *Eulaema meriana* and *Eu*. *bombiformis*, were present on all nine plateaus and so were considered widespread (Fig 2). Four other species were absent on only one plateau (*Euglossa augaspis*, *E*. *chalybeata*, *E*. *imperialis* and *Eu*. *mocsary*). Altogether, these six species form the set of core species for the sampled plateaus (15%). On the other hand, 24 species were classified as rare (or singleton species) since they had scattered distributions (e.g., *Euglossa* sp. n., *Eufriesea vidua*), and were collected only on one (13 species) or two (11 species) plateaus. Two species, *Euglossa ioprosopa* and *E*. *decorata*, had somewhat disjunctive distributions, occurring on plateaus 30 km distant from each other.

**Fig 4:**
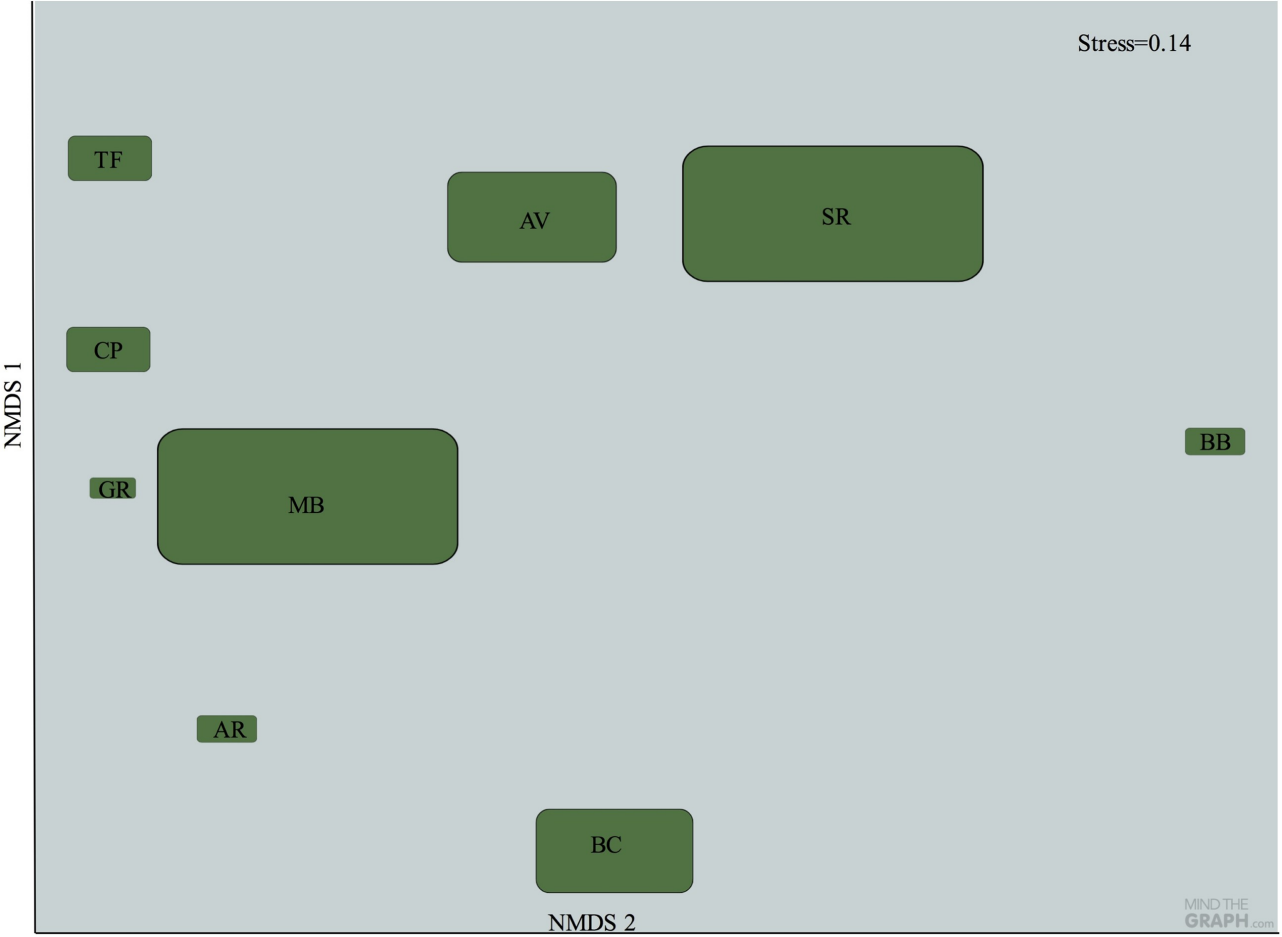
Non-metric multidimensional scaling (NMDS) ordination of the 9 sampling sites according to their similarity in Orchid bee species composition (Simpson index for presence or absence data). Site codes are in methods section and in Fig 1. Size of the shapes represent the proportional size of the plateaus.

The two dimensional NMDS (stress 0.14, proportion of variance 0.70) showed the existence of a geographical/environmental gradient in orchid bee species composition among the plateaus (Table 1 and Fig 4). Axis 1 depicts a longitudinal gradient in temperature, with the plateaus on the right side of the ordination plot 193 (BB) having low average temperature, high evapotranspiration and only one dry month, whereas axis 2 described a gradient in rainfall 195 seasonality. In particular, axis 2 discriminated plateaus AV and SR from the others (Fig 4), based on their high average temperature and 2–3 dry months with the consequent low average rainfall.

**Table 1:** Pearson correlations between the environmental and geographic variables and the non-metric multidimensional scaling (NMDS) ordination scores (for a two-dimensional ordination of the nine sampling sites according to their similarity in orchid bee species composition).

Variables	Axis 1	Axis 2
Average Temperature	0,55873**	-0,53571**
Number of dry months	-0,37966	0,49041*
Precipitation	0,13123	-0,57939**

*p<0.05

**p<0.01

**Fig 5:**
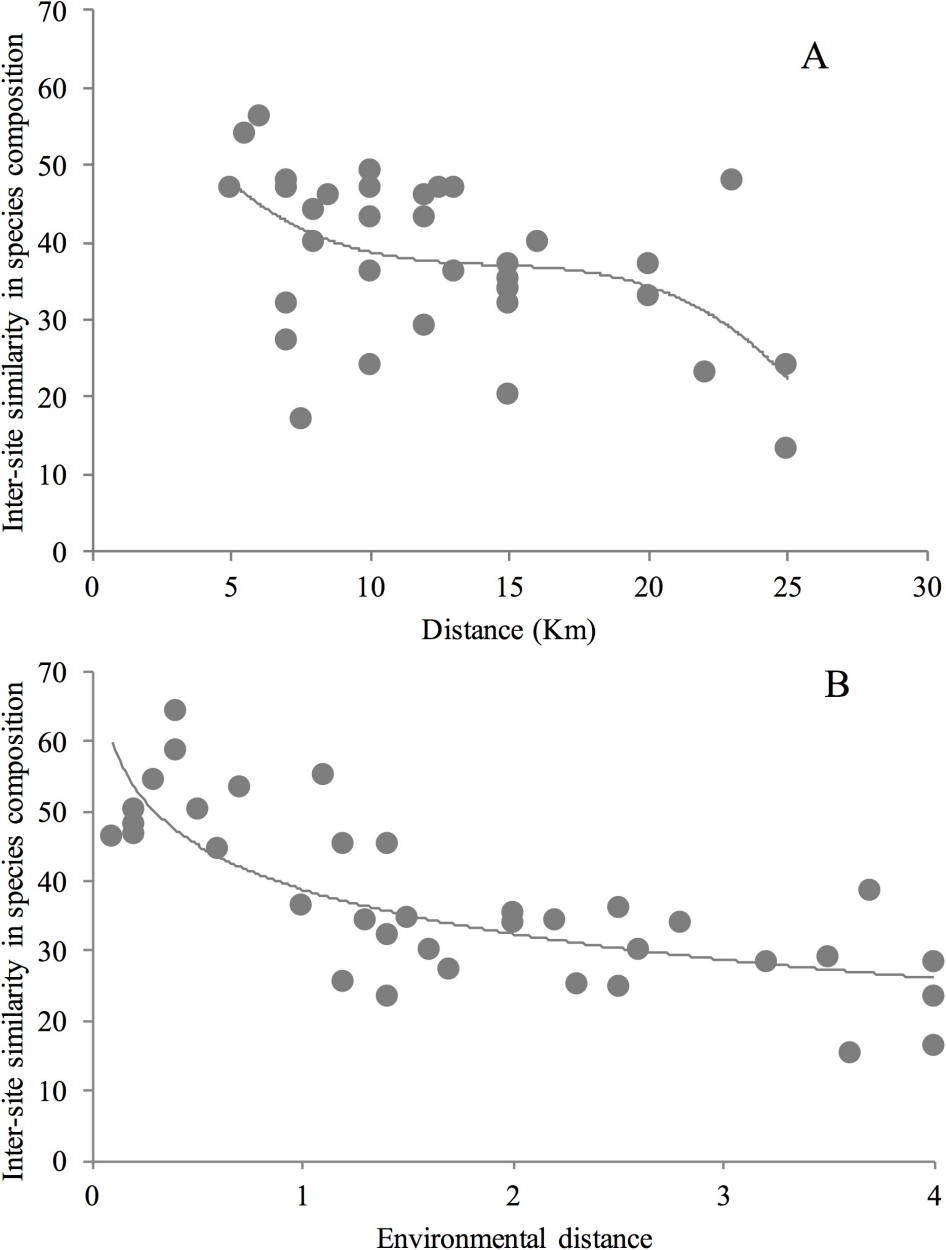
Decay in the similarity of orchid-bee assemblages in relation to the geographic A or B environmental distance between sampling sites. Geographic distance is the distance in km between paired sites. Environmental distance is the Euclidean distance between paired sites with respect to three climatic variables (average annual temperature, average annual precipitation, and precipitation seasonality). Similarity in orchid-bee species composition is based on the Bray–Curtis Index of similarity. Lines represent the logarithmic regression curve.

The level of pairwise similarity among the nine bee communities sampled was highly variable, ranging from 20.0 to 61.1% (Bray-Curtis Index, mean = 40.7%) when considering the total abundance of the 30 most frequent species. Similar results were obtained when considering the presence or absence of all 55 recorded species (Jaccard index: mean = 40.3%, range = 17.0–70.3%). Similarity in orchid bee species composition among the different pairs of plateaus decayed both as function of climatic (R = -0.34, p = 0.03) (Fig 5A) and geographic distances among sites (R = -0.53, p<0.001; Fig 5B). The greatest dissimilarities in bee assemblage composition were observed among pairs of sites located 20–30 km from each other (Monte Branco and Bacaba) (see Fig 1). There was a positive and statistically significant relationship between the number of trees and orchid bee species richness (R2 = 0.48, p = 0.036).

**Fig 6:**
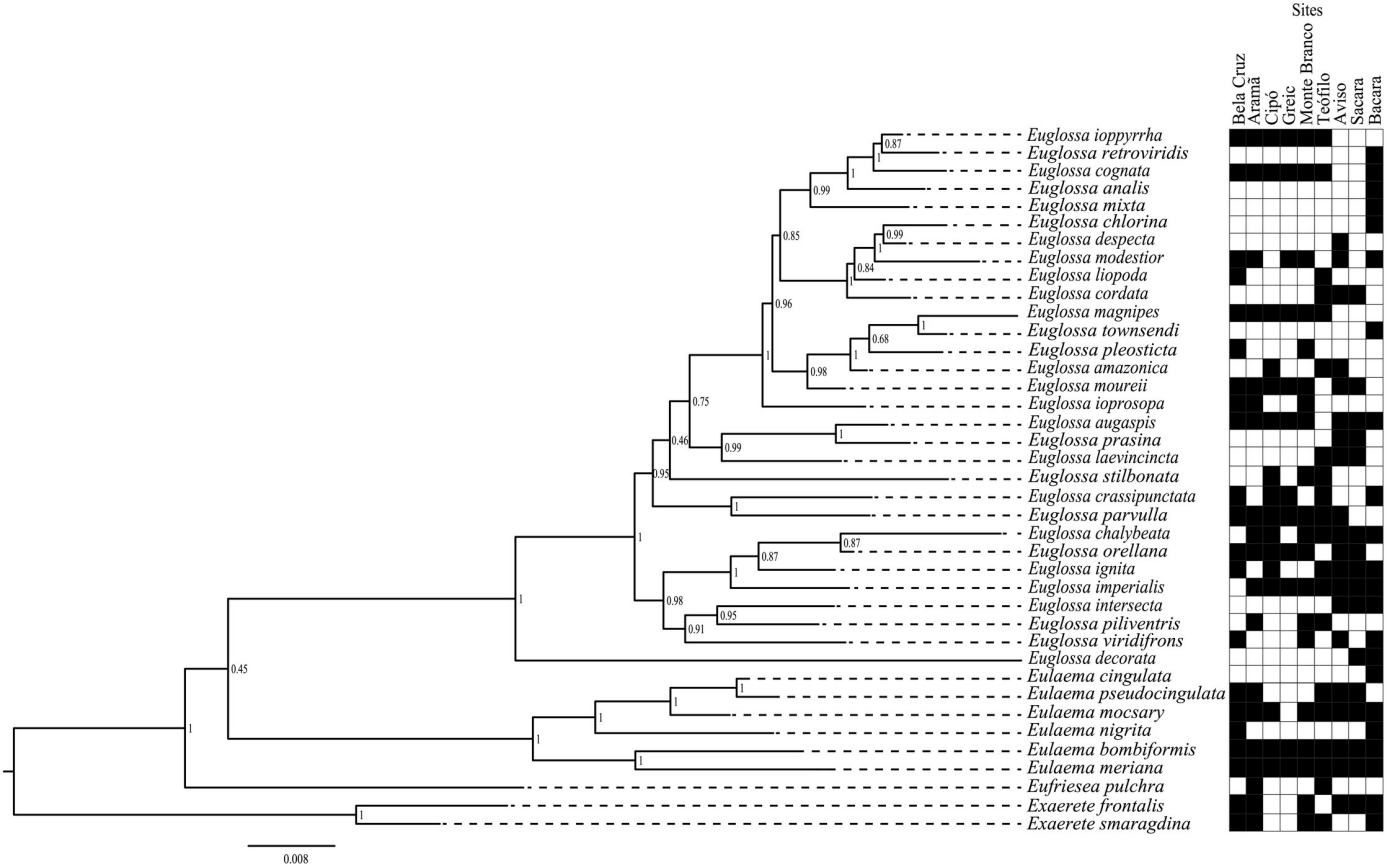
Bayesian phylogenetic analysis based on mitochondrial (COI) and nuclear (EF1-α, Argk, and Poll-II) loci, and occurrence site (black square) for each orchid bee species. Node numbers indicates posterior probabilities.

We estimated a Bayesian tree for orchid bees sampled in the present study from multigene dataset (Fig 6). Most of the relationships among species were highly supported and agreed with the phylogeny previously proposed for the group [35].

**Fig 7:**
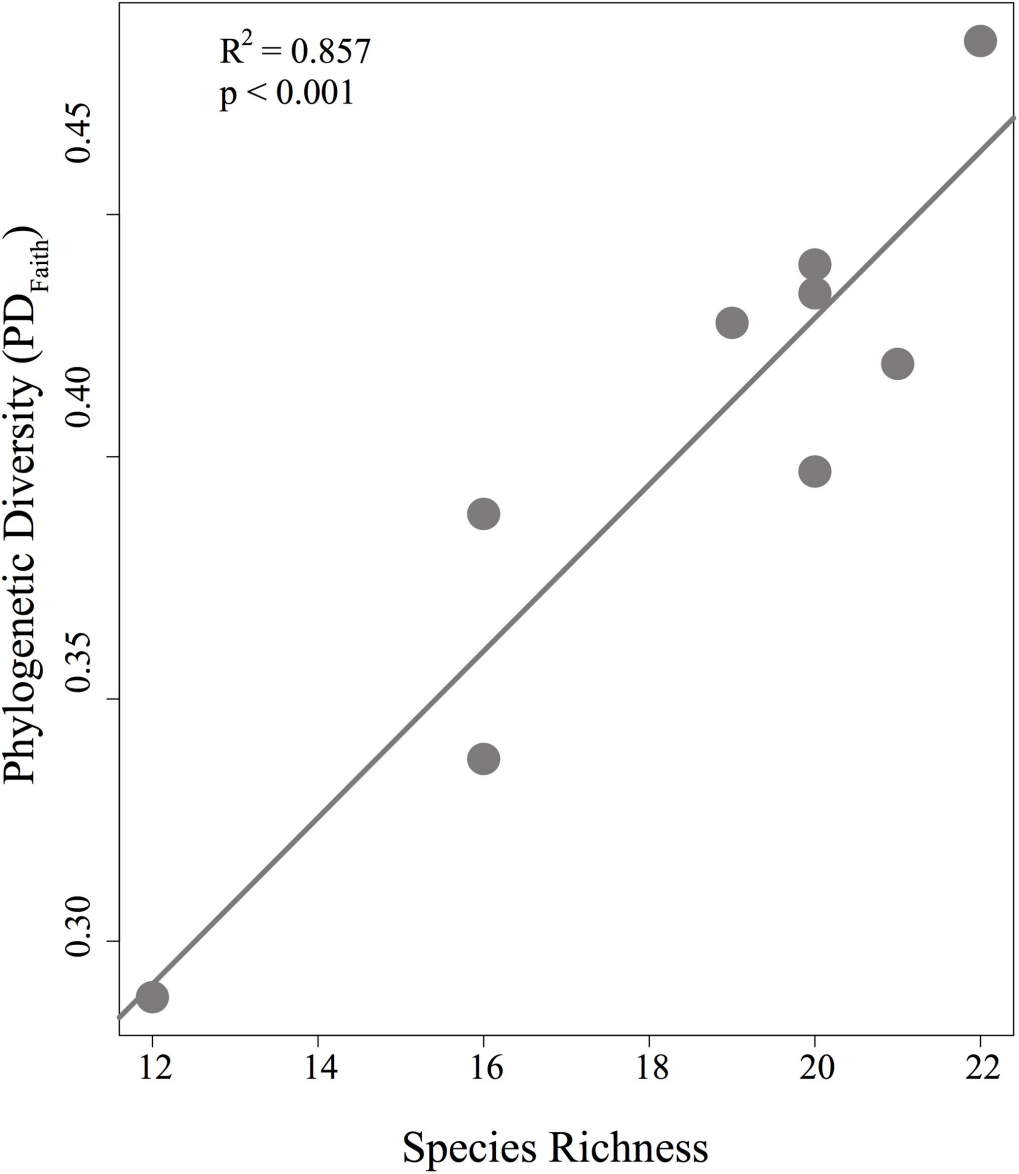
Relationship between phylogenetic diversity (PD) and species richness. Dots represent each plateau.

We examined how patterns of phylogenetic diversity varied across different plateaus. Phylogenetic diversity (PD_Faith_ and MPD) were lowest in the Greig plateau (0.288 and 0.064), whereas Bacaba plateau hold the higher PD_Faith_ (0.486) and Monte Branco plateau showed the highest MPD value (0.082) (Table 2). PD_Faith_ patterns, but not MPD patterns, were highly congruent with bee species richness (R2 = 0.857, p < 0.001; Fig 7). The relationships between phylogenetic diversity (both metrics) and plateau size were not significant (S1 Fig).

**Table 2:** Comparison of two phylogenetic diversity metrics (PD_Faith_ and MPD) from multiloci phylogeny of orchid bee. The values are given for each site.

Sites	PD	MPD
Bela Cruz	0.419	0.079
Aramã	0.428	0.076
Cipó	0.338	0.074
Greig	0.288	0.070
Monte Branco	0.434	0.082
Teófilo	0.440	0.073
Aviso	0.397	0.071
Sacará	0.388	0.070
Bacaba	0.486	0.077

PD_Faith_: Faith’s phylogenetic diversity

MPD: mean phylogenetic distance

## Discussion

The species richness of orchid bees observed among the plateaus in the Amazonian Rainforest of Saracá-Taquera, Brazil, 55 species, is rivaled only by that observed in Tarapoto, Peru (50 species) [27] and central Panama [42]. Furthermore, species richness of FLONA Saracá-Taquera has no known parallel in the Amazon Basin, with orchid-bee samplings in the central and western Amazon of Brazil typically recording from 16 to 38 species [43–46; 23–25]. Furthermore, the region of FLONA Saracá-Taquera harbors many species that are rare and endemic to the Amazon Basin [47]; only the most abundant species were widely distributed on all plateaus.

This study reports the occurrence of a large number of singletons (25%) at FLONA Saracá-Taquera, indicating that the communities of orchid bees are not characterized by species that are abundant and dominant for a given locality. In fact, most orchid bee species have relatively small distributions, and only 12 species are known to occur throughout southern Mexico to southeastern Brazil [21]. This is extremely important from a conservationist perspective Forest destruction could lead these species to extinction.

Our findings provide further support to the hypothesis that both climate and spatial factors can explain a substantial amount of the variation in beta-diversity (regional-scale species richness) over large geographic areas see [14]. The geographic distribution of individual bee species may be determined by a number of factors such as climate, vegetation structure, and competition with similar species [48], as well as resource availability, such as nesting sites, resin, pollen, nectar and perhaps even microbial mutualists [49]. The plateau with the richest orchid bee community in this study also had the highest species richness of trees, which may provide the resources necessary to support orchid bee populations. Although orchid bees are known to play a key role in pollination of forest plants, detailed knowledge of their natural history is still lacking; yet forest destruction by mining is advancing without proper scientific studies on natural history and sound governance by environmental agencies in the region.

In general, as we anticipated, similarity in bee assemblage composition was inversely proportional to the distance between plateaus, yet we did find some high similarity between some distantly separated pairs of plateaus. For instance, although the Aramã and Monte Branco plateaus were separated by more than 30 km of valleys, they shared 88% of their species composition (the most common species). This observation is reinforced by the low rate of distance decay in community similarity and by the relatively large number of singleton species. High similarity over long distances has also been reported for Amazonian herbivorous insect communities [50–51] and ant communities [5]. These authors claimed that high community similarity over long distances can be a result of incomplete sampling and the consequent omission of rare endemic species. However, as in other studies with highly diverse tropical insect assemblages [51, 5], presence or absence of rare insect species did not affect our results. Many Amazonian orchid bees appear to be habitat generalists e.g [21,25, 27,45], which may help to explain why a relatively large number of the species we collected exhibited a broad distribution across the study area.

Our results are in accordance with many other studies conducted in the Amazon Basin in showing that environmental gradients strongly affect the turnover of animal and plant species in tropical forests ants [5], orchid bees [6, 52], plant species [53–54]. However, geographic distance among plateaus explained almost twice as much of the observed differences in the orchid bee data set as did climatic information. On the other hand, the climatic information explained variation that was not spatially structured, indicating that within each study area, the species and the climatic data had similar spatial arrangements [55] or that climatic variation was too little to be responsible for explaining, the community distribution. In fact, part of the observed differences in community composition with increasing distance appears not to be explained by a concomitant gradient in precipitation.

The size of the forest plateaus adequately predicted the richness of orchid bees in the FLONA Saracá-Taquera region, since larger plateaus had higher species richness. Although both PD and MPD were no significantly related to the plateau size (S1 Fig), lesser PDFaith and MPD were observed in the smaller and less diverse plateau (Greig–Table 2).

Concordantly, the larger plateau (Bacaba) showed the highest PDFaith, and it was expected because this metric is closest related to species richness (Fig 7). However, Monte Branco showed the highest MPD value, likely because MPD was weighted by abundance, and the abundant species in this community were very distantly related. However, because MPD counts each branch of the phylogenetic tree multiple times depending on the number of species in a community, there are some concerns on its use [56].

In general, these data provide support for the contention that forest plateaus in the Amazon region behave as islands and that they experience processes peculiar to them, at least when orchid bees are considered. Likewise, greater abundance and richness of orchid bee species in larger forest fragments were reported by 315 Storck-Tonon et al. [25] in the Amazon, [57] and in southern Costa Rica. Also, Nemésio & Silveira [58] argued that orchid bee abundance tends to increase with fragment size in southeastern Brazil, although no correlation between species richness and fragment size was observed.

The crucial factors in determining diversity, composition, and temporal variability of pollinator assemblages are climatic seasonality and spatial and temporal variation in food resources. Although orchid bee species are specialized pollinators of orchids, their distribution and assemblages are also influenced by the structure and composition of trees in the habitat [21,59], which may explain the positive relationship between tree richness and orchid bee species richness. Most biological communities appear to be structured by both local ecological determinism and dispersal-driven assembly. According to Dick et al. [60], the absence of geographical structure in Amazonian orchid bees may also derive from Quaternary population expansion. However, there is no convincing evidence for a major change in forest cover in the Amazon during the glacial periods of the Pleistocene [60–61], which might explain recent population expansion in the Euglossine taxa. The occurrence of a high number of co-generic Euglossine species suggests that this region has experienced a history of active speciation as a result of the high heterogeneity of habitats available. Recent population expansion may result from this recent speciation, associated to high levels of gene flow, past or present, across the Neotropical lowlands, which may explain the greater diversity found in FLONA Saracá- Taquera, as well as the higher species richness on each plateau. For example, evidence for extensive gene flow across the Amazon Basin would suggest a small role for regional endemic diversification, and therefore low levels of species turnover across broad spatial scales for some taxa.

Little is known about seasonal changes of different pollinator guilds at a given locality. In particular, studies along climatic gradients in tropical systems are completely lacking. Studies considering single groups of pollinators suggest 340 that the seasonal variation in species richness of butterflies as well as of bees and wasps [21, 35, 62–63] are more pronounced than in hummingbirds, because insect pollinators can outlive phases of unfavorable environmental conditions in larval stages or by hibernating as adults [6]. For instance, a greater number of species of insect pollinators during the dry season was recorded when rain did not restrict their flight activities [35]. Besides, Euglossine bee’s species all include some that are highly seasonal, but others that breed continuously [35].

Although located inside a national forest, species survival is not guaranteed as this Forest was created with the goal to protect not just the natural resources but also the right of a company to mine them [64]. Since species with wide regional distributions are less subject to extinction [65], we argue that the orchid bee fauna of the plateaus of FLONA Saracá-Taquera is endangered because only few species were found to be widely distributed. As suggested by D.W. Roubik (pers. info.) the 50 across all sites, considering the low abundance of many, may mean that a lot of extinction is fairly recent, but not necessarily caused by humans. This concern is extremely worrisome given the interest in the extraction of the bauxite of the plateaus, which would decimate them, probably with irreversible impacts on biodiversity and ecosystem loss.

## Supporting information

S1 Fig Regression analyses relating phylogenetic diversity (PD (A), and MPD (B) to plateau size in hectare. Dots represent each plateau.
(TIF)

S1 Table Collection sites, with abundance information, for orchid bee species in the study area and Genbank accession numbers. (DOCX)

S2 Table Models of nucleotide evolution for each gene were determined with jModeltest 2.1.4 using the Bayesian information criterion. (DOC)

S3 Table Richness and abundance of Euglossine bees in each of the nine plateaus. (DOCX)
