# Supplementary figures and images for: Patterns of orchid bee species diversity and turnover among forested plateaus of central Amazonia
Source: PLoS One. 2017 Apr 14;12(4):e0175884. doi: 10.1371/journal.pone.0175884 (PMC5391963; doi:10.1371/journal.pone.0175884)

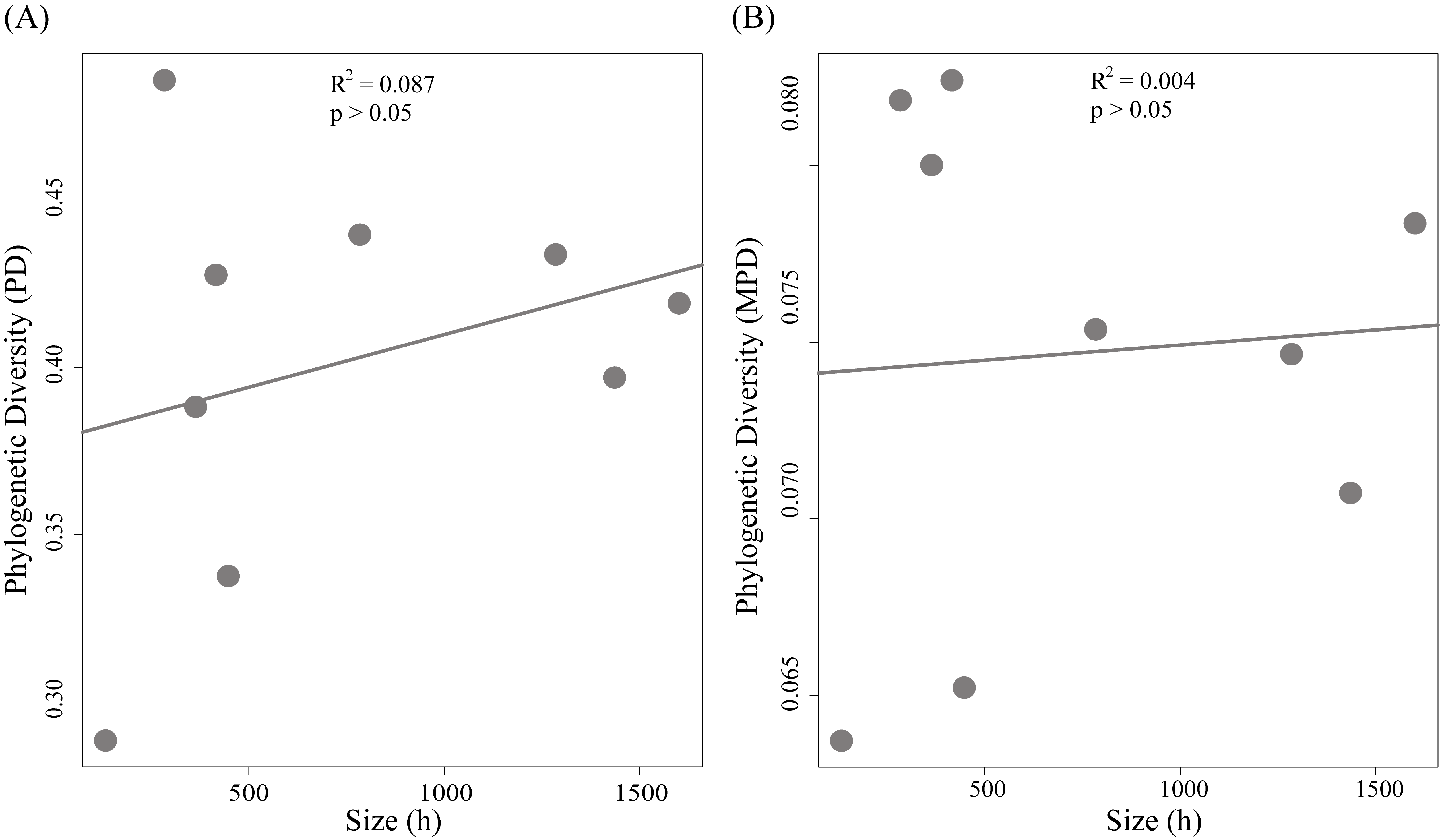

Supplement: S1 Fig — Regression analyses relating phylogenetic diversity (PD (A), and MPD (B) to plateau size in hectare. Dots represent each plateau. (TIF) [file pone.0175884.s001.tif]
