# Supplementary material for: Patterns of orchid bee species diversity and turnover among forested plateaus of central Amazonia
Source: PLoS One. 2017 Apr 14;12(4):e0175884. doi: 10.1371/journal.pone.0175884 (PMC5391963; doi:10.1371/journal.pone.0175884)
Supplement: S1 Table — (DOCX) [file pone.0175884.s002.docx]

S1 Table: Models of nucleotide evolution for each gene were determined with jModeltest 2.1.4 using the Bayesian information criterion.

| Gene | Model of nucleotide evolution |
| --- | --- |
| COI | GTR+I+G |
| ArgK | HKY+G |
| Pol-II | HKY+I |
| EF1-α | HKY+G |
