# Supplementary material for: Patterns of orchid bee species diversity and turnover among forested plateaus of central Amazonia
Source: PLoS One. 2017 Apr 14;12(4):e0175884. doi: 10.1371/journal.pone.0175884 (PMC5391963; doi:10.1371/journal.pone.0175884)
Supplement: S2 Table — (DOC) [file pone.0175884.s003.doc]

Supplementary material:

Table S1: Collection sites, with abundance information, for orchid bee species in the study area and Genbank accession numbers.

|  | Collected sites | | | | | | | | | Loci access numbers (Genbank) | | | |
| --- | --- | --- | --- | --- | --- | --- | --- | --- | --- | --- | --- | --- | --- |
| Species | BC | AR | CP | GR | MB | TF | AV | SR | BA | COI | ArgK | Poll-II | EF1-α |
| **Eufriesea laniventris* | 2 | 2 | 0 | 0 | 0 | 0 | 0 | 0 | 1 | - | - | - | - |
| *Eufriesea pulchra* | 0 | 1 | 0 | 0 | 0 | 1 | 0 | 0 | 0 | EU421506 | EU421637 | EU421257 | EU421377 |
| **Eufriesea vidua* | 0 | 0 | 0 | 0 | 0 | 0 | 0 | 0 | 5 | - | - | - | - |
| *Euglossa amazonica* | 0 | 0 | 1 | 0 | 0 | 2 | 1 | 0 | 0 | EU421466 | EU421594 | EU421218 | EU421335 |
| *Euglossa analis* | 0 | 0 | 0 | 0 | 0 | 0 | 0 | 0 | 4 | EU421545 | EU421674 | EU421292 | EU421417 |
| *Euglossa augaspis* | 10 | 18 | 7 | 7 | 13 | 0 | 10 | 2 | 35 | EU421519 | EU421650 | EU421267 | EU421391 |
| **Euglossa avicula* | 41 | 31 | 15 | 7 | 90 | 10 | 13 | 0 | 0 | - | - | - | - |
| *Euglossa chalybeata* | 0 | 2 | 1 | 0 | 2 | 2 | 15 | 15 | 50 | EU421513 | EU421644 | - | EU421385 |
| *Euglossa chlorine* | 0 | 0 | 0 | 0 | 0 | 0 | 0 | 0 | 1 | EU421525 | EU421656 | EU421272 | EU421396 |
| *Euglossa cognata* | 5 | 8 | 8 | 5 | 2 | 1 | 0 | 0 | 14 | EU421510 | EU421640 | EU421261 | EU421381 |
| *Euglossa cordata* | 0 | 0 | 0 | 0 | 0 | 1 | 3 | 0 | 0 | EU421469 | EU421597 | EU421221 | EU421338 |
| *Euglossa* aff. *cordata* | 0 | 0 | 0 | 0 | 0 | 0 | 0 | 1 | 0 | EU421469 | EU421597 | EU421221 | EU421338 |
| *Euglossa crassipunctata* | 13 | 0 | 12 | 4 | 0 | 13 | 0 | 0 | 8 | EU421494 | EU421626 | EU421246 | EU421365 |
| *Euglossa decorata* | 0 | 0 | 0 | 0 | 0 | 0 | 0 | 2 | 1 | EU421504 | EU421636 | EU421255 | EU421375 |
| *Euglossa despecta* | 0 | 0 | 0 | 0 | 0 | 0 | 1 | 0 | 0 | EU421568 | EU421696 | EU421314 | EU421441 |
| **Euglossa. gaianii* | 6 | 0 | 8 | 3 | 1 | 2 | 1 | 2 | 0 | - | - | - | - |
| *Euglossa ignita* | 4 | 0 | 1 | 0 | 0 | 10 | 1 | 2 | 35 | EU421490 | EU421621 | EU421241 | EU421360 |
| *Euglossa imperialis* | 0 | 3 | 2 | 4 | 3 | 9 | 1 | 1 | 27 | EU421537 | EU421668 | EU421284 | EU421408 |
| *Euglossa intersecta* | 0 | 0 | 0 | 0 | 0 | 0 | 8 | 2 | 3 | EU421503 | EU421635 | - | EU421374 |
| *Euglossa ioppyrrha* | 10 | 10 | 6 | 5 | 2 | 1 | 0 | 0 | 0 | EU421470 | EU421599 | EU421222 | EU421340 |
| *Euglossa ioprosopa* | 2 | 1 | 0 | 0 | 2 | 0 | 0 | 0 | 0 | EU421523 | EU421654 | EU421270 | EU421394 |
| *Euglossa laevicincta* | 0 | 0 | 0 | 0 | 0 | 1 | 1 | 1 | 0 | EU421527 | EU421658 | EU421274 | EU421398 |
| *Euglossa liopoda* | 1 | 0 | 0 | 0 | 0 | 4 | 0 | 0 | 0 | EU421566 | EU421694 | EU421312 | EU421439 |
| *Euglossa magnipes* | 12 | 3 | 14 | 12 | 13 | 13 | 0 | 0 | 0 | JQ844896 | - | - | - |
| *Euglossa mixta* | 0 | 0 | 0 | 0 | 0 | 0 | 0 | 0 | 2 | - | - | EU421309 | EU421436 |
| *Euglossa modestior* | 1 | 4 | 0 | 2 | 4 | 0 | 1 | 0 | 7 | EU421541 | EU421672 | EU421288 | EU421412 |
| *Euglossa moureii* | 2 | 2 | 1 | 1 | 1 | 0 | 1 | 1 | 0 | JQ844897 | - | - | - |
| *Euglossa orellana* | 4 | 9 | 2 | 3 | 6 | 0 | 8 | 15 | 0 | - | EU421643 | EU421263 | EU421384 |
| *Euglossa parvula* | 12 | 2 | 4 | 15 | 10 | 5 | 6 | 0 | 0 | EU421564 | EU421692 | EU421310 | EU421437 |
| *Euglossa piliventris* | 0 | 1 | 0 | 0 | 1 | 1 | 0 | 0 | 0 | - | EU421624 | EU421244 | EU421363 |
| *Euglossa pleosticta* | 4 | 0 | 0 | 0 | 7 | 0 | 0 | 0 | 0 | JQ844899 | - | - | - |
| *Euglossa prasina* | 0 | 0 | 0 | 0 | 0 | 0 | 1 | 1 | 0 | EU421563 | EU421691 | EU421308 | EU421435 |
| *Euglossa retroviridis* | 0 | 0 | 0 | 0 | 0 | 0 | 0 | 0 | 1 | EU421571 | EU421699 | EU421317 | EU421444 |
| *Euglossa stilbonata* | 0 | 0 | 1 | 0 | 2 | 2 | 0 | 0 | 0 | EU421509 | - | EU421260 | EU421380 |
| *Euglossa townsendi* | 0 | 0 | 0 | 0 | 0 | 0 | 0 | 0 | 47 | JQ844900 | - | - | - |
| **Euglossa.* cf*. violaceifrons* | 8 | 12 | 9 | 14 | 7 | 5 | 0 | 0 | 0 | - | - | - | - |
| *Euglossa viridifrons* | 3 | 0 | 0 | 0 | 5 | 0 | 1 | 0 | 1 | EU421476 | EU421605 | - | EU421346 |
| **Euglossa* sp. n. | 0 | 0 | 0 | 0 | 0 | 0 | 1 | 0 | 0 | - | - | - | - |
| **Euglossa (Glossura)* sp.1 | 0 | 0 | 0 | 0 | 0 | 5 | 0 | 0 | 2 | - | - | - | - |
| **Euglossa (Glossura)* sp.2 | 0 | 0 | 0 | 0 | 0 | 0 | 0 | 0 | 36 | - | - | - | - |
| **Euglossa (Euglossa)* sp.1 | 0 | 0 | 0 | 0 | 0 | 0 | 0 | 0 | 3 | - | - | - | - |
| **Euglossa (Glossurela)* sp.1 | 0 | 0 | 0 | 0 | 0 | 0 | 0 | 0 | 2 | - | - | - | - |
| **Euglossa (Glossura)* sp.3 | 0 | 0 | 0 | 0 | 0 | 0 | 0 | 0 | 2 | - | - | - | - |
| **Euglossa (Glossura)* sp.4 | 0 | 0 | 0 | 0 | 0 | 0 | 0 | 0 | 1 | - | - | - | - |
| **Euglossa (Glossura)* sp. 5 | 0 | 0 | 0 | 0 | 0 | 0 | 0 | 0 | 1 | - | - | - | - |
| **Euglossa (Glossura)* sp.6 | 0 | 0 | 0 | 0 | 0 | 0 | 0 | 0 | 1 | - | - | - | - |
| **Euglossa (Euglossa)* sp.2 | 0 | 0 | 0 | 0 | 0 | 0 | 0 | 0 | 1 | - | - | - | - |
| *Eulaema bombiformis* | 23 | 21 | 12 | 16 | 9 | 9 | 10 | 5 | 6 | EU421524 | EU421655 | EU421271 | EU421395 |
| *Eulaema cingulata* | 0 | 0 | 0 | 0 | 0 | 0 | 0 | 0 | 13 | EU421501 | EU421633 | EU421253 | EU421372 |
| *Eulaema meriana* | 31 | 18 | 26 | 10 | 16 | 4 | 59 | 52 | 87 | EU421512 | EU421642 | EU421262 | EU421383 |
| *Eulaema mocsary* | 22 | 2 | 4 | 0 | 12 | 1 | 8 | 7 | 7 | EU421502 | EU421634 | EU421254 | EU421373 |
| *Eulaema nigrita* | 1 | 0 | 0 | 0 | 0 | 0 | 0 | 0 | 1 | EU421555 | EU421685 | EU421300 | EU421427 |
| *Eulaema pseudocingulata* | 4 | 2 | 0 | 0 | 0 | 11 | 9 | 6 | 0 | JQ844901 | - | - | - |
| *Exaerete frontalis* | 2 | 1 | 0 | 0 | 10 | 0 | 1 | 3 | 13 | EU421478 | EU421607 | - | EU421352 |
| *Exaerete smaragdina* | 9 | 3 | 0 | 0 | 4 | 5 | 0 | 0 | 6 | EU421457 | EU421585 | EU421211 | EU421326 |

*Species without genetic information.

Site names - BC: Bela Cruz; AR: Aramã; CP: Cipó; GR: Greig; MB: Monte Branco; TF: Teófilo; AV: Aviso; SR: Saracá; BA: Bacaba.
