# Supplementary material for: Patterns of orchid bee species diversity and turnover among forested plateaus of central Amazonia
Source: PLoS One. 2017 Apr 14;12(4):e0175884. doi: 10.1371/journal.pone.0175884 (PMC5391963; doi:10.1371/journal.pone.0175884)
Supplement: S3 Table — (DOCX) [file pone.0175884.s004.docx]

| Bee species | Plateau names | | | | | | | | | |
| --- | --- | --- | --- | --- | --- | --- | --- | --- | --- | --- |
|  | Aramã | Aviso | Bacaba | Bela Cruz | Cipó | Greic | Monte Branco | Saraca | Teofilo |  |
| *Eufrisea laeiventris* | 2 | 0 | 1 | 2 | 0 | 0 | 0 | 0 | 0 |  |
| *Eufrisea pulchra* | 1 | 0 | 0 | 0 | 0 | 0 | 0 | 0 | 1 |  |
| *Eufrisea vidua* | 0 | 0 | 5 | 0 | 0 | 0 | 0 | 0 | 0 |  |
| *Euglossa (Euglossa)* sp.1 | 0 | 0 | 3 | 0 | 0 | 0 | 0 | 0 | 0 |  |
| *Euglossa (Euglossa)* sp.2 | 0 | 0 | 1 | 0 | 0 | 0 | 0 | 0 | 0 |  |
| *Euglossa (Glossura)* sp.1 | 0 | 0 | 2 | 0 | 0 | 0 | 0 | 0 | 5 |  |
| *Euglossa (Glossura)* sp.2 | 0 | 0 | 36 | 0 | 0 | 0 | 0 | 0 | 0 |  |
| *Euglossa (Glossura)* sp.3 | 0 | 0 | 2 | 0 | 0 | 0 | 0 | 0 | 0 |  |
| *Euglossa (Glossura)* sp.4 | 0 | 0 | 1 | 0 | 0 | 0 | 0 | 0 | 0 |  |
| *Euglossa (Glossura)* sp.5 | 0 | 0 | 1 | 0 | 0 | 0 | 0 | 0 | 0 |  |
| *Euglossa (Glossura)* sp.6 | 0 | 0 | 1 | 0 | 0 | 0 | 0 | 0 | 0 |  |
| *Euglossa (Glossurela)* sp.1 | 0 | 0 | 2 | 0 | 0 | 0 | 0 | 0 | 0 |  |
| *Euglossa amazonica* | 0 | 1 | 0 | 0 | 1 | 0 | 0 | 0 | 2 |  |
| *Euglossa analis* | 0 | 0 | 4 | 0 | 0 | 0 | 0 | 0 | 0 |  |
| *Euglossa augaspis* | 18 | 10 | 35 | 10 | 7 | 7 | 13 | 2 | 0 |  |
| *Euglossa avicula* | 31 | 13 | 0 | 41 | 15 | 7 | 90 | 0 | 10 |  |
| *Euglossa chalybeata* | 2 | 15 | 50 | 0 | 1 | 0 | 2 | 15 | 2 |  |
| *Euglossa chlorina* | 0 | 0 | 1 | 0 | 0 | 0 | 0 | 0 | 0 |  |
| *Euglossa congnata* | 8 | 0 | 14 | 5 | 8 | 5 | 2 | 0 | 1 |  |
| *Euglossa cordata* | 0 | 3 | 0 | 0 | 0 | 0 | 0 | 0 | 1 |  |
| *Euglossa cordata* | 0 | 0 | 0 | 0 | 0 | 0 | 0 | 1 | 0 |  |
| *Euglossa crassipunctata* | 0 | 0 | 8 | 13 | 12 | 4 | 0 | 0 | 13 |  |
| *Euglossa decorata* | 0 | 0 | 1 | 0 | 0 | 0 | 0 | 2 | 0 |  |
| *Euglossa despecta* | 0 | 1 | 0 | 0 | 0 | 0 | 0 | 0 | 0 |  |
| *Euglossa gaianii* | 0 | 1 | 0 | 6 | 8 | 3 | 1 | 2 | 2 |  |
| *Euglossa ignita* | 0 | 1 | 35 | 4 | 1 | 0 | 0 | 2 | 10 |  |
| *Euglossa imperiallis* | 3 | 1 | 27 | 0 | 2 | 4 | 3 | 1 | 9 |  |
| *Euglossa intersecta* | 0 | 8 | 3 | 0 | 0 | 0 | 0 | 2 | 0 |  |
| *Euglossa iopphyrra* | 10 | 0 | 0 | 10 | 6 | 5 | 2 | 0 | 1 |  |
| *Euglossa ioprosopa* | 1 | 0 | 0 | 2 | 0 | 0 | 2 | 0 | 0 |  |
| *Euglossa irisa* | 0 | 1 | 0 | 0 | 0 | 0 | 0 | 0 | 0 |  |
| *Euglossa laevicincta* | 0 | 1 | 0 | 0 | 0 | 0 | 0 | 1 | 1 |  |
| *Euglossa liopoda* | 0 | 0 | 0 | 1 | 0 | 0 | 0 | 0 | 4 |  |
| *Euglossa magnipes* | 3 | 0 | 0 | 12 | 14 | 12 | 13 | 0 | 13 |  |
| *Euglossa mixta* | 0 | 0 | 2 | 0 | 0 | 0 | 0 | 0 | 0 |  |
| *Euglossa modestior* | 4 | 1 | 7 | 1 | 0 | 2 | 4 | 0 | 0 |  |
| *Euglossa moureii* | 2 | 1 | 0 | 2 | 1 | 1 | 1 | 1 | 0 |  |
| *Euglossa orellana* | 9 | 8 | 0 | 4 | 2 | 3 | 6 | 15 | 0 |  |
| *Euglossa parvula* | 2 | 6 | 0 | 12 | 4 | 15 | 10 | 0 | 5 |  |
| *Euglossa piliventris* | 1 | 0 | 0 | 0 | 0 | 0 | 1 | 0 | 1 |  |
| *Euglossa pleostica* | 0 | 0 | 0 | 4 | 0 | 0 | 7 | 0 | 0 |  |
| *Euglossa prasina* | 0 | 1 | 0 | 0 | 0 | 0 | 0 | 1 | 0 |  |
| *Euglossa retroviridis* | 0 | 0 | 1 | 0 | 0 | 0 | 0 | 0 | 0 |  |
| *Euglossa stilbonata* | 0 | 0 | 0 | 0 | 1 | 0 | 2 | 0 | 2 |  |
| *Euglossa towsendii* | 0 | 0 | 47 | 0 | 0 | 0 | 0 | 0 | 0 |  |
| *Euglossa violaceifrons* | 12 | 0 | 0 | 8 | 9 | 14 | 7 | 0 | 5 |  |
| *Euglossa viridifrons* | 0 | 1 | 1 | 3 | 0 | 0 | 5 | 0 | 0 |  |
| *Eulaema bombiformis* | 21 | 10 | 6 | 23 | 12 | 16 | 9 | 5 | 9 |  |
| *Eulaema cingulata* | 0 | 0 | 13 | 0 | 0 | 0 | 0 | 0 | 0 |  |
| *Eulaema meriana* | 18 | 59 | 87 | 31 | 26 | 10 | 16 | 52 | 4 |  |
| *Eulaema mocsaryi* | 2 | 8 | 7 | 22 | 4 | 0 | 12 | 7 | 1 |  |
| *Eulaema nigrita* | 0 | 0 | 1 | 1 | 0 | 0 | 0 | 0 | 0 |  |
| *Eulaema pseudocingulata* | 2 | 9 | 0 | 4 | 0 | 0 | 0 | 6 | 11 |  |
| *Exaerete frontalis* | 1 | 1 | 13 | 2 | 0 | 0 | 10 | 3 | 0 |  |
| *Exaerete smaragdina* | 3 | 0 | 6 | 9 | 0 | 0 | 4 | 0 | 5 |  |
